# Supplementary material for: Structure-Based Screening and Optimization of PafA Inhibitors with Potent Anti-Tuberculosis Activity
Source: Int J Mol Sci. 2024 Dec 8;25(23):13189. doi: 10.3390/ijms252313189 (PMC11642889; doi:10.3390/ijms252313189)
Supplement: Supplementary file 1 [file ijms-25-13189-s001.zip › Supplementary Figure.pdf]

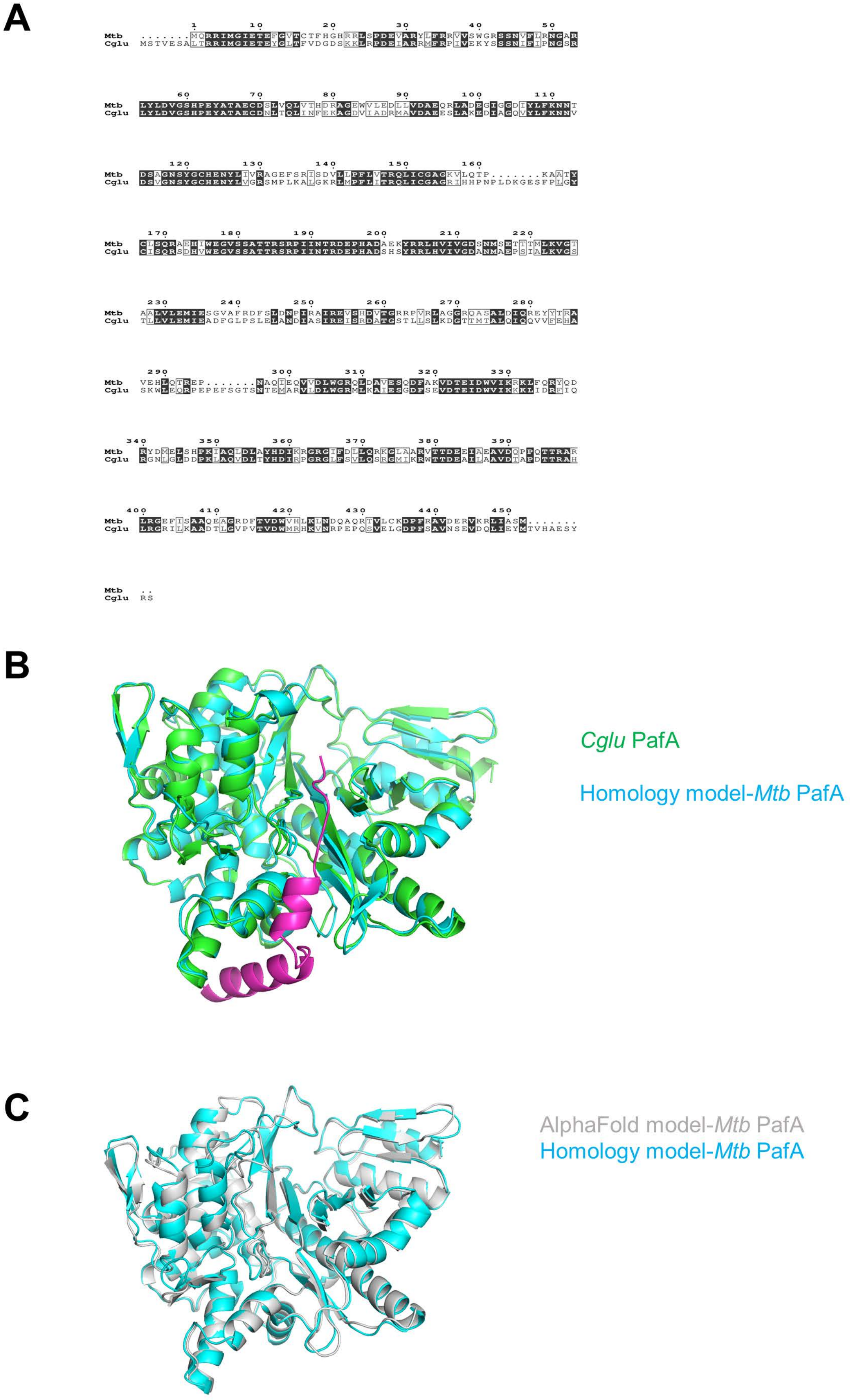

Figure S1 ***Mtb*** **PafA 3D structure based on homology modeling using the crystal structure** ***Cglu*** **PafA (PDB ID: 4BJR)**

Figure S1. *Mtb* PafA 3D structure based on homology modeling using the crystal structure of *Cglu* PafA (PDB ID: 4BJR). (**A**) Alignment of *Mtb* PafA to *Cglu* PafA. Sequences were compiled from the National Center for Biotechnology Information server and aligned by means of ClustalW. (**B**) Superimposition of *Cglu* PafA (PDB:4BJR, green, Pup is colored in magenta) and homology modeling of *Mtb* PafA (cyan) structures. (**C**) Superimposition of AlphaFold modeling (grey) and homology modeling (cyan) of *Mtb* PafA structures**.**

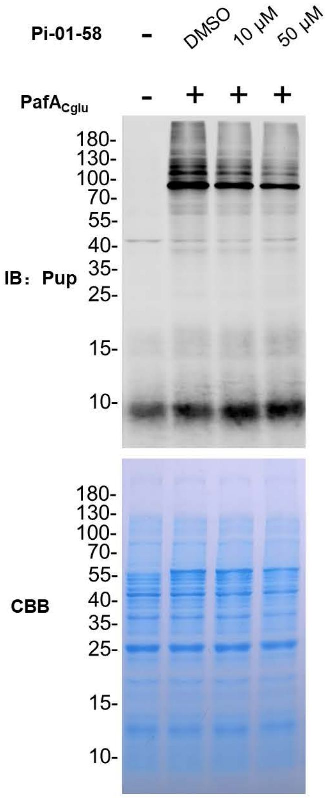

Fig. S2 *Cglu* PafA activity is inhibited by Pi-1-58

Figure S2. *Cglu* PafA activity is inhibited by Pi-1-58. *E. coli* total protein pupylation activity of *Cglu* PafA with Pi-1-58. Samples were analyzed by SDS-PAGE, followed by CBB staining to serve as a loading control and Western blotting with an anti-Pup antibody.

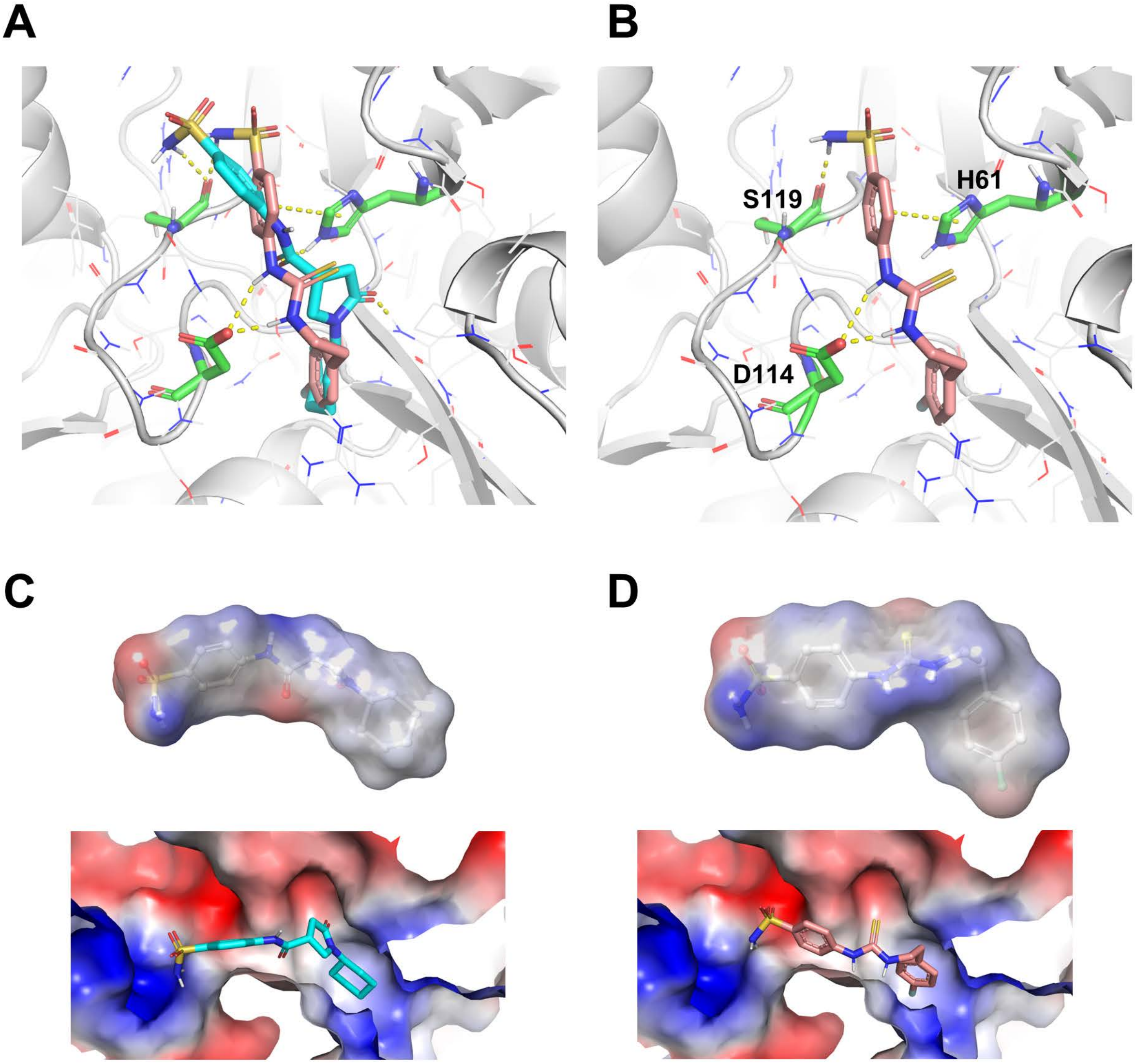

Fig. S3 PafA inhibitor Pi-2-26 derived from the computer aided inhibitor screening assay

Figure S3. PafA inhibitor Pi-2-26 derived from the computer-aided inhibitor screening assay. (**A**) Superimposition of the modeled Pi-1-58 (cyan stick) and Pi-2-26 (salmon stick) complex. (**B**) Interaction details between Pi-2-26 (salmon stick) and PafA (white cartoon). Residues within 6 Å of Pi-2-26 are shown as white lines. Interacting residues are shown as green sticks and labeled. (**C**) Electrostatic potential surfaces of Pi-1-58 and the binding sites of *Mtb* PafA. (**D**) Electrostatic potential surfaces of Pi-2-26 and the binding sites of *Mtb* PafA.

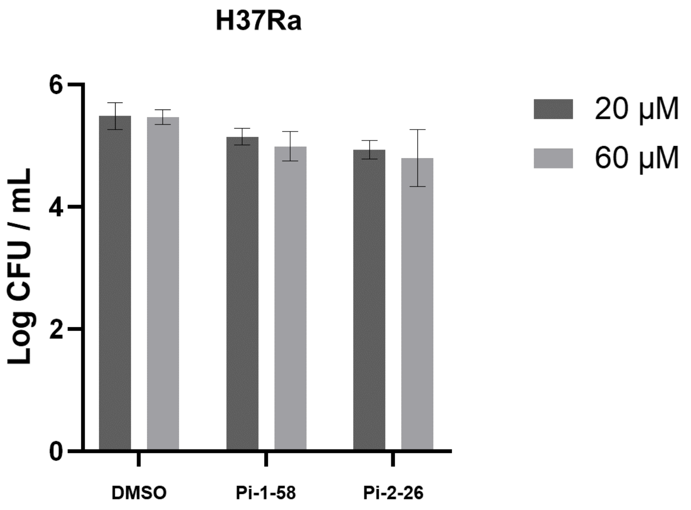

Fig. S4 Nitric oxide stress assays on *Mtb* with PafA inhibitors.

Figure S4. Killing of *Mtb* H37Ra strains in Sauton's medium with PafA inhibitors. Killing of *Mtb* H37Ra strains in Sauton's medium. *Mtb* H37Ra strains are treated with Pi or DMSO at the indicated concentrations.
